# Comparison of interdental papillae around single implants in the tissue-level (TL) and bone-level (BL) implants: A clinical trial

**DOI:** 10.34172/japid.2020.007

**Published:** 2020-05-19

**Authors:** Seyed Ahmad Banihashem Rad, Ali Forouzanfar, Seyed Ali Banihashemrad

**Affiliations:** ^1^Student Research Committee, School of Dentistry, Mashhad University of Medical Sciences, Mashhad, Iran; ^2^Department of Periodontics, School of Dentistry, Mashhad University of Medical Science, Mashhad, Iran

**Keywords:** Dental Implants, Interproximal Soft Tissue, Interproximal Papilla

## Abstract

**Background:**

This study aimed to evaluate the effect of bone-level implants in comparison with tissue-level implants on dental papilla dimensions in single-tooth implants.

**Methods:**

In the present clinical trial, 50 patients, 24 females (48%) and 26 males (52%), were selected among patients requesting single implants in the posterior area of the jaws. The subjects were divided into two groups (n=25). The subjects in the first group were treated with a bone-level implant (Implantium, Dentium, Korea), and the subjects in the second group were treated with a tissue level implant (Implantium, Dentium, Korea). None of the implants were loaded during this period, and only the healing effect was measured. All the implants underwent one-stage surgery (none-submerged), and healing abutments were placed after implantation. The papilla heights in both the mesial and distal aspects of the adjacent teeth were measured. A periodontal probe was used to measure from the top of the papilla to the CEJ of the adjacent teeth in two time intervals. Descriptive statistics were performed using tables and Shapiro-Wilk, chi-squared, Mann-Whitney, and independent t-tests.

**Results:**

The findings showed that the interdental papilla in TL single implants performed better than that in BL implants at the three-month interval. This difference was statistically significant on the mesial aspect but not on the distal aspect. However, the difference was not clinically significant.

**Conclusion:**

A comparison of papilla dimensions in two implant types showed that papilla formation in TL implants was better than that in BL implants at the three-month postoperative interval.

## Introduction

Aesthetics has become an important issue in contemporary implant dentistry in the last decade.^1^ Successful therapy can no longer be judged by whether the implants osseointegrate or not.^2^ Esthetic appearance can be strongly influenced by the tissues surrounding the implant. Gingival facial appearance and interproximal papilla are the most vital elements that make favorable soft tissue.^3^ Notably, some aesthetic assessment grades have been proposed for peri-implant soft tissue outcomes.^4^ In addition, several authors have suggested surgical modifications and different loading protocols to achieve the best soft tissue integration.^5^ This is especially more challenging in compromised sites, caused by trauma, atrophy, periodontal disease, and/or infection.^6^

The interproximal space depends on several components that can compromise the interdental papilla. Inappropriate contours of restorations or prosthetic crowns, abnormal tooth shape, traumatic flossing habits, and interproximal hygiene procedures, and more importantly, periodontal diseases can cause recession in the interdental papilla.^7^ The marginal bone level of the peri-implant and soft tissue is firmly connected, which determines the aesthetic outcome.^8^

Although the exact etiology of crestal bone changes around dental implants has not yet entirely been elucidated, many factors have been suggested to inﬂuence this situation.^9,10^ Among all the factors affecting this phenomenon, the implant type (one-piece vs. two-piece), the abutment type (platform switching or matching platform—i.e., with or without a horizontal offset), the location of the implant–abutment junction regarding the crest of the alveolar bone, and the stability of the adjacent tooth.^11,12^

Some researches believe that the supracrestal position of implant placement results in signiﬁcantly less marginal bone reactions as compared to crestally placed implants.^13^ The present study was designed to assess the effect of bone-level implants in comparison with tissue-level implants on dental papilla dimensions in single-tooth implants. In a general classification, implants are categorized into two groups, bone-level implants and tissue-level implants.

In the present prospective clinical trial, the records of all the patients undergoing implant procedures for the replacement of posterior teeth using Implantium bone-level implants (BL) and Implantium tissue-level implants (TE), from January 10th, 2018 to December 1st, 2019 in the Department of Periodontology, Mashhad University of Medical Science were evaluated. With bone-level implants, the platform is placed at the level of the jawbones. Bone-level implants must be inserted deep enough into the bone so as not to show the metallic surfaces. These implants must be placed in the esthetic zone. However, tissue-level implants usually are covered with a collar with a flat titanium surface. The platforms of TL implants are generally located 1.5–3 mm superior to the BL implants. However, since the posterior zones of both arches are not in the esthetic zone and are not visible during smiling and speaking, TL implants should be used in the non-esthetic zone. Another bright side of TL implants is that they can be inserted with single-stage surgery, with no further need for a second surgical procedure. This causes the peri-implant soft tissues with sufficient time and opportunity for regeneration, development, and stability. To sum up, BL implants are used in both the esthetic and non-esthetic zones, but TL implants are appropriate only in the non-esthetic zone.^14^

In this survey, since we wanted to compare these two implant types, all the dental implants were placed in the posterior regions and were single-tooth implants. Studies have shown verities in the level of the papilla in the mesial and distal aspects around single implant-supported restorations. The distal papilla has a lower score in the Jemt^15^ classification compared to the mesial papilla adjacent to tooth surfaces.^16^ Jemt reported that during a 1‒3-year period after single-implant restorations, the adjacent papilla regenerates spontaneously. The reason for spontaneous papilla recovery is not apparent, but it might be suggested that the inflammation is due to plaque accumulation in the proximal space, leading to soft tissue swelling.^17^

## Methods

**Figure 1:**
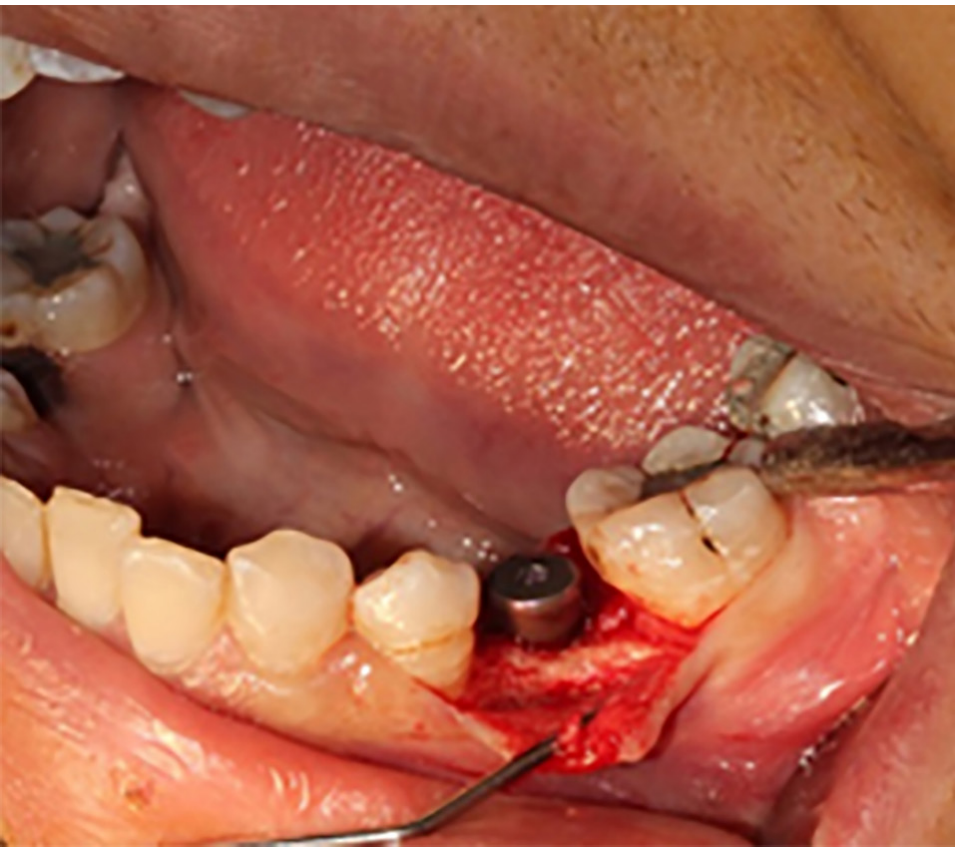
The healing abutment is placed after implant placement (one-stage surgery).

**Figure 2:**
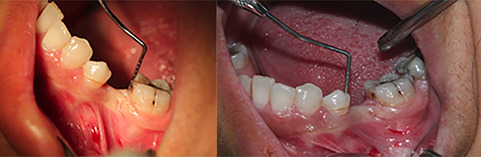
Measurement of dental papilla height in the mesial and distal aspects before surgery.

Fifty patients, 24 women (48%) and 26 men (52%), were selected for the present clinical trial among patients requiring single implants in the posterior areas of the jaws. The subjects were divided into two groups (n=25). The first group was treated with bone-level implants (Implantium, Dentium, Korea), and the second group was treated with tissue-level implants (Implantium, Dentium, Korea). A sealed envelope system coded as (A, B) was used in this survey for double-blinding of the study, in which neither the patient nor the clinician was aware of which treatment the patient was randomized to. Half of the patients included in the study received BL implants, and the other half received TL implants. In all the patients, all the surgical procedures were completed by one periodontist and under standard conditions at the center of the tooth loss area concerning the future prosthetic considerations. All the implants underwent one-stage surgery (none-submerged), and the healing abutments were placed after implant placement (Figure 1). None of the implants were loaded during this period, and only the healing effect was measured. The patients were evaluated for papillary dimensions in two mesial and distal dimensions in two groups (n=25) of BL and TL at two time intervals before and three months after implant placement (Figure 2). The measurements of papilla height in both the mesial and distal aspects of the adjacent teeth were measured. A standard periodontal probe was used to measure from the top of the papilla to the CEJ of the adjacent tooth at two time intervals (Figure 2).

The body surface of Implantium implants is composed of SLA (sandblast large-grit acid etch), and the body thread is a buttress. Microthreads are present in the neck of the Implantium system.

The inclusion criteria of the patients consisted of no systemic disease, no pregnancy, no smoking, and the presence of suitable soft and hard tissue without the need for hard and soft tissue augmentation, presence of a posterior single-tooth vacancy, an interest in implant treatment, absence of periodontal diseases, proper oral hygiene, no pocket depths >3 mm, no bone loss, a safe distance from the adjacent tooth (at least 1.5 mm), thick gingival biotype, no history of radiotherapy and chemotherapy. The exclusion criteria consisted of uncontrolled active systemic disease, the need for bone grafts (GBR), thin gingival biotype, and prolonged steroid therapy.

Descriptive statistics were reported using tables and analyzed using chi-squared and Mann-Whitney tests and independent t-test. The level of significance was set at P<0.05.

## Results

In this study, 50 participants (24 females (48%) and 26 males (52%), with a mean age of 40.36 ±10.49 years and an age range of 24–60 years, were evaluated. Patients were evaluated for papillary dimensions in both the mesial and distal aspects in two groups (n=25) of bone-level (BL) and tissue-level (TL) implants at two time intervals before and three months after implantation.

Table 1 presents the numbers, means, standard deviations, minimums, maximums, medians, and quadratic variables of BL and TL groups and statistical test results.As can be seen in both the BL and TL groups, the means of both the mesial and distal variables increased significantly three months postoperatively compared to the baseline.

**Table 1:** Comparison of the variables before and three months after surgery

**Group**	**Variable**	**Number**	**Mean**	**S.D**	**±MedianInterquartile range**	**Wilcoxon test result**
**BL**	**Mesial before surgery**	25	2.58	0.77	3.0 ± 1.00	Z=3.81 P<0.001
	**Mesial 3month after surgery**	25	3.22	0.69	3.5 ± 0.50	
	**Distal before surgery**	25	2.40	0.54	2.5 ± 1.00	Z=3.80 P<0.001
	**Distal 3month after surgery**	25	3.02	0.71	3.0 ± 1.25	
**TL**	**Mesial before surgery**	25	2.60	0.43	2.5 ± 1.00	Z=4.33 P<0.001
	**Mesial 3month after surgery**	25	3.64	0.55	4.0 ± 0.50	
	**Distal before surgery**	25	2.48	0.62	2.5 ± 1.00	Z=4.16 P<0.001
	**Distal 3month after surgery**	25	3.32	0.64	3.5 ± 0.75	

Table 2 depicts the numbers, means, standard deviations, minimums, maximum, medians, and quadratic variables. As shown in Table 2, the medians and interquartile ranges in the mesial aspect of the BL and TL groups before surgery were 1.00±3.00 and 1.00±2.50, respectively, with no significant difference (P=0.645). The medians and interquartile ranges of the distal aspect before surgery in the BL and TL groups were 1.50±2.50 and 1.00±2.50, respectively, with a significant difference (P=0.498). The medians and interquartile ranges in the BL and TL groups were 3.50±0.50 and 0.50±4.00, respectively, with a significant difference (P=0.011). The medians and interquartile ranges of mesial changes three months after surgery were 1.00±0.50 and 1.00±1.00 in the BL and TL groups, respectively, with a significant difference (P=0.013).

**Table 2:** Comparison of the variables between BL and TL

**variable**	**Group**	**Number**	**Mean**	**S.D**	**Min**	**Max**	**±Median Interquartile range**	**Yu-Mann Whitney test results**
**Mesial before surgery**	**BL**	25	2.58	0.77	1.0	4.0	3.0 ± 1.00	Z=0.46 P=0.645
	**TL**	25	2.60	0.43	2.0	3.0	2.5 ± 1.00	
**Distal before surgery**	**BL**	25	2.40	0.54	1.5	3.5	2.5 ± 1.00	Z=0.68 P=0.498
	**TL**	25	2.48	0.62	1.0	3.5	2.5 ± 1.00	
**Mesial 3month after surgery**	**BL**	25	3.22	0.69	1.5	4.5	3.5 ± 0.50	Z=2.53 P=0.011
	**TL**	25	3.64	0.55	2.0	4.5	4.0 ± 0.50	
**Distal 3month after surgery**	**BL**	25	3.02	0.71	1.5	4.0	3.0 ± 1.25	Z=1.46 P=0.144
	**TL**	25	3.32	0.64	2.0	4.5	3.5 ± 0.75	
**Mesial change**	**BL**	25	0.64	0.49	0.0	1.5	0.5 ± 1.00	Z=2.48 P=0.013
	**TL**	25	1.04	0.52	0.0	2.0	1.0 ± 1.00	
**Distal change**	**BL**	25	0.62	0.56	0.0	2.0	0.5 ± 1.00	Z=1.55 P=0.120
	**TL**	25	0.84	0.53	0.0	2.0	1.0 ± 0.75	

In the following section, the crucial variables of age and gender are compared between the two groups.

### Age

According to Table 3, the age range (the highest and lowest age difference) was higher in the BL group compared to the TL group.

**Table 3:** Statistical indices of mean, standard deviation, minimum, maximum of the age variable in the BL and TL groups

**Group**	**Number**	**Mean**	**S.D**	**Min**	**Max**	**Independent t-test result**
** BL**	25	39.48	10.13	24	60	T=0.59 P=0.558
** TL**	25	41.24	10.97	25	60	

The mean age in the BL group was 39.4810±10.13, with 41.24±10.97 years in the TL group, with no significant difference (P=0.558).

### Gender

According to Table 4, the number of males in each BL and TL group was 13 (52%); however, the number of females in each BL and TL group was 12 (48%). The gender distribution in the study groups was quite similar (P=1.00).

**Table 4:** Gender distribution in study groups

**Group**		**Gender**		**All**
		**Female**	**Male**	
**BL**	**Number**	12	13	25
	**Percentage**	48.0%	52.0%	100.0%
**TL**	**Number**	12	13	25
	**Percentage**	48.0%	52.0%	100.0%
**All**	**Number**	24	26	50
	**Percentage**	48.0%	52.0%	100.0%
**Chi-squared test result**				X^2^=0.00 P=1.00

**Figure 3:**
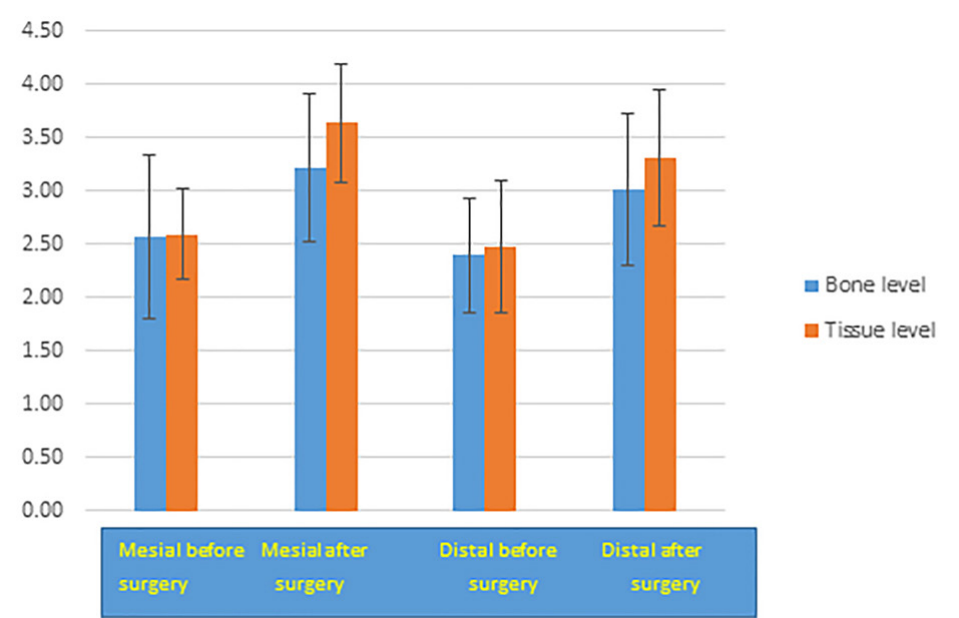
Comparison of mean and SD (standard deviation).

The data presented in Table 4 can be seen more clearly in Figure 3. According to these data, the growth and formation of distal papilla were less than that of the mesial papilla, and its dimension was less than that on the mesial aspect even before implant placement.

## Discussion

Replacing missing teeth with implants is a successful treatment option. However, currently, the main issue is not the osseointegration of dental implants. Successful implant treatment means gaining the best esthetic outcomes, in addition to the stability and function of the implant.^18^

In this study, interdental papillae were compared in two different commonly used implant types (BL and TL from the Implantium system). The findings showed that interdental papilla in TL single implants grew better than the BL at the three-month interval. This difference was statistically significant on the mesial aspect but not on the distal aspect. However, this difference might not be clinically significant.

The strength of this study, in comparison with other studies, was the attention to interdental papilla as a critical element in the esthetic outcomes and success of implants.

It should be noted that most of the studies did not consider our subject, and a small number of studies, in which interdental papilla around different designs of implants was compared, had designs and variables different from those of the present study. Therefore, those studies cannot be directly compared with the present study.

Chang et al^16^ made a comparative assessment of soft issue dimensions between single-tooth implants and a contralateral natural tooth. They measured the papilla dimension in 20 patients treated with a single tooth implant using the Jemt index. The follow-up period in this study was 38 months on average. The smaller papilla formation on the distal side and its smaller size than the mesial papilla in the present study are consistent with the results of this study. This is an interesting observation, suggesting that the anatomy of the adjacent tooth, (e.g., the diameter of the root, the proximal outline/curvature of the cementoenamel junction, connective tissue attachment, in addition to the amount of mesiodistal space between implant and tooth, might significantly affect the papilla dimensions between the tooth and implant.

In a retrospective cohort study in 2014, Kumar et al^18^ compared the amount of marginal bone loss (MBL) between a bone-level and a soft tissue-level implant system with similar intra-bony shape and surface composition. Implant depth of insertion was measured in all the patients. Measurements were made at three intervals of 12, 24, 36 months. They concluded that BL implants had significantly less bone loss than TL implants in 12 months. There was also a significant relationship between the depth of implant insertion and the amount of bone loss, which was higher in deeply inserted implants. There was no statistically significant difference between the two groups in the 6–12-month period. In the present study, the rate of papilla formation in the three-month interval was higher in the TL implants than in the BL implants, which was only significant on the mesial aspect, suggesting that better inter-dental papilla formation could be due to lower bone loss around these implants. However, this difference was not clinically significant. A more favorable formation of papilla on the mesial aspect of the present study might be because of better oral hygiene on the mesial aspect of the implant.

Roccuzzo et al^19^ performed a systematic review to study the formation of papilla between the tooth and implant regarding the space between the level of interproximal bone and the prosthetic contact point. The vertical distance varied between 2 and 11 mm, and the range of papilla fill (Jemt’s score of 2–3) was between 56.5% and 100% of cases.

There is some proof that the vertical distance from the base of the contact point to the crest of bone level affects the interproximal papilla dimension; i.e., the lower is the distance, the higher is the percentage of papilla fill. Thorough embrasure fill between an implant and the tooth is dependent on the integrity of the periodontal ligament of the tooth. To decrease the chance of aesthetic failures, interproximal probing on the adjacent teeth should be evaluated before implant insertion. In the current study, the Jemt index was not used, but the papilla height was measure precisely in mm.

Vincent et al^20^ conducted a study to determine whether there is any connection between the distance from the base of the contact point to the bone crest and the regeneration of interproximal papilla, and also whether the surgical technique at uncovering affects the results.

They performed a clinical and radiographic retrospective assessment of the level of papilla around single dental implants and their adjacent teeth in the anterior maxilla in 26 patients restored with 27 implants. Six months after implant placement, 17 implants were uncovered with a standard technique, while 10 implants were uncovered with another technique, which is meant to generate papilla-like formation around dental implants. They showed that when the distance from the contact point to the bone crest was ≤5, the papilla was regenerated almost all the time. When the distance was ≥6 mm, the papilla was detectable in 50% of the cases or less. They also indicated a positive effect for the modified surgical technique, aimed at reconstructing the papilla at the implant uncovering.

The usual surgical procedure involves a crestal incision with a small releasing incision described by Odman^21^ and Adell.^22^ However, the modified surgical procedure described by Adriaenssens^23^ by augmenting soft tissue to create papilla has resulted in better papilla formation. In the present study, the usual surgical procedure was performed by a periodontist, and it is recommended that the modified technique be used and compared with the routine procedures.

Goiat et al^24^ surveyed a better perception of factors that could result in papilla formation or recession, such as the type of zone where the implant was placed. They conducted a systematic review of the literature respecting the generation or recession of papilla near the implants inserted in fresh, healing, or healed zones. They reported that the sites where the implants were inserted did not have a long-term effect on papilla formation or recession. It is more vital to think of other factors, such as the initial condition of the patient’s age and the distance between the implant and the adjacent tooth. In the present study, all the implants were inserted in the posterior mandible and maxilla, and since they all were single-tooth implants, variations in the sites would not damage the results.

Gastaldo et al^25^ conducted a survey to evaluate the influence of the vertical and horizontal distances between adjacent implants and also between teeth and implants in the presence of interproximal papilla. They reported that the best distance from the base of the contact point to the bone crest between adjacent implants is 3 mm and 3–5 mm between a tooth and an implant. The best lateral spacing between implants and between a tooth and an implant is 3–4 mm. Moreover, there is an interaction between the horizontal and vertical distances when the lateral spacing is >3 mm.

Si et al^26^ studied changes in the papilla next to single-tooth implant restorations in the esthetic zone in the anterior maxilla after placing the crown and by considering the impact of soft tissue thickness on the formation of the papilla. The patients were divided into two sub-groups based on the mucosal thickness: group 1, 1.5 mm≤mucosal thickness≤3 mm; and group 2, 3 mm<mucosal thickness≤4.5 mm. Two prosthodontists performed a comparison of the interproximal papillae at the time of crown placement (baseline) and six months after loading (follow-up) by using the papilla fill index (PFI). They reported that the interproximal papilla level around single-implant restorations could regenerate significantly over time six months after restoration in terms of PFI assessments. The thicker mucosa before implant placement gave rise to a better esthetic outcome in papilla changes. In that study, only patients with thick mucosa were included, and the outcomes cannot be directly compared with the present study.

The limitations of the current survey include the small and insufficient sample size and short follow-up period. More extensive clinical trials are definitively required with a larger sample size to gain more clinical data. The periodontist in the present was very expert with all the delivered interventions, and this could limit the extrapolation of the results of the present study, but all the procedures were carried out under clinical conditions.

## Conclusion

The results indicated that interdental papilla in TL single implants performed better than BL at the three-month interval with a statistically significant difference in the mesial aspect and no significant difference in the distal aspect. However, this difference was not clinically significant.

## Authors’ contributions

AF presented the concept of the study. SALBR and SAHBR de-signed the study. All the authors were responsible for the intellectual content of the study. SAHBR conducted the clinical study. The manuscript was prepared and edited by SALBR. SAHBR reviewed the manuscript.

## Competing Interests

The authors declare no conflict of interest.

## Ethics Approval

All the patients signed a consent form prior to surgery. This study was approved in Mashhad Dental School Ethics Committee under the code IR.MUMS.DENTISTRY.REC.1398.101.
